# Effects of Vitamin D Restricted Diet Administered during Perinatal and Postnatal Periods on the Penis of Wistar Rats

**DOI:** 10.1155/2018/6030646

**Published:** 2018-04-23

**Authors:** Flávia Fernandes-Lima, Bianca M. Gregório, Fernanda A. M. Nascimento, Waldemar S. Costa, Carla B. M. Gallo, Francisco J. B. Sampaio

**Affiliations:** ^1^Urogenital Research Unit, State University of Rio de Janeiro, Rio de Janeiro, RJ, Brazil; ^2^Federal University of Rio de Janeiro, Rio de Janeiro, RJ, Brazil

## Abstract

Vitamin D deficiency is common in pregnant women and infants. The present study aimed to investigate the effects of vitamin D restricted diet on the* Wistar* rats offspring penis morphology. Mother rats received either standard diet (SC) or vitamin D restricted (VitD) diet. At birth, offspring were divided into SC/SC (from SC mothers, fed with SC diet) and VitD/VitD (from VitD mothers, fed with VitD diet). After euthanasia the penises were processed for histomorphometric analysis. The VitD/VitD offspring displayed metabolic changes and reduction in the cross-sectional area of the penis, corpus cavernosum, tunica albuginea, and increased area of the corpus spongiosum. The connective tissue, smooth muscle, and cell proliferation percentages were greater in the corpus cavernosum and corpus spongiosum in the VitD/VitD offspring. The percentages of sinusoidal spaces and elastic fibers in the corpus cavernosum decreased. The elastic fibers in the tunica albuginea of the corpus spongiosum in the VitD/VitD offspring were reduced. Vitamin D restriction during perinatal and postnatal periods induced metabolic and structural changes and represented important risk factors for erectile dysfunction in the penis of the adult offspring. These findings suggest that vitamin D is an important micronutrient in maintaining the cytoarchitecture of the penis.

## 1. Introduction

Vitamin D deficiency in pregnant women and infants is a global health problem [1]. Low maternal serum vitamin D levels is associated with adverse outcomes of pregnancy such as intrauterine growth restriction and neonatal low birth weight [2].

Micronutrients such as many vitamins regulate the expression of key genes involved in growth and proliferation and in the functional attributes of specific organs [3]. The literature points out that the micronutrient imbalance may also affect the development of the androgen-dependent organs, such as penis [4].

The formation of the penis is dependent on androgens hormones during the development of the reproductive system. In addition, androgens must act during the late fetal period and/or postnatal period to foster growth of the penis [5].

Vit D acts on calcium and phosphorus homeostasis, as well as on bone metabolism, and performs other extra bone functions [6–8]. The active form of vit D is produced primarily in the kidneys and circulates in the blood and binds to its receptor present in the cells of almost all tissues [9]. Currently, vit D is considered a potent steroid hormone that promotes effects on the various organs, including the penis [10, 11].

The biological activities of vit D are mediated by its receptor (VDR). VDR and the enzymes that metabolize vit D are expressed in the testis, sperm, epididymis, seminal vesicle, prostate, and kidney, which indicates the importance of this vitamin in the urogenital system and in reproduction [12, 13]. Vit D is considered an essential micronutrient, important for maintaining vital functions, including penile erection [14].

Vit D deficiency is associated with atherogenic dyslipidemia, diabetes mellitus, and reduced serum testosterone levels that are associated with endothelial dysfunction and are classic risk factors for the onset of erectile dysfunction [15]. These metabolic changes are associated with venous leakage of the corpus cavernosum, with damage to endothelial cells, and with decreased production of nitric oxide (NO), which is essential for maintaining erection [15, 16]. Vit D deficiency stimulates the renin-angiotensin system, which may increase the expression of angiotensin II. This induces inflammatory response and vascular smooth muscle hypertrophy. In addition, vit D regulates the synthesis of endothelial nitric oxide synthase and NO [16].

Reduction of NO production prevents vasodilation and causes atherosclerosis by promoting vasoconstriction, vascular smooth muscle growth, decreased fibrinolysis, and thrombosis [17, 18].

Hypovitaminosis D accentuates risk factors for cardiovascular diseases and may lead to erectile dysfunction (ED) [19, 20]. It is possible to make an association of ED with the first symptoms of atherosclerosis and to even point ED as one of the possible predictors of cardiovascular diseases [21, 22].

Barassi et al. (2014) showed that patients with ED have vit D deficiency and that this condition is more frequent in patients with atherogenic etiology for ED [23]. Low levels of vit D may increase the risk of ED by promoting endothelial dysfunction [23].

Vit D has antiproliferative influence on smooth muscle cells which indicates antiatherosclerotic properties, protecting from ED [24, 25].

Vit D deficiency is associated with reduced serum levels of testosterone. Individuals with vit D deficiency presented reduced serum testosterone, increased thickness of the cavernous artery tunica, decreased artery flow, and decreased erectile function [15].

With regard to the rodent penis, used for our study, it is known that they present different morphology from the human penis. The trabeculae of the corpora cavernosa are the main tissue structures of the penis involved in erection. They are composed of smooth and endothelial muscle cells, an extracellular matrix composed of collagen, elastic system fibers, and sinusoidal spaces [26, 27]. These spaces are filled by blood for intumescence and penile rigidity during erection.

The corpus cavernous of the rat differs from that of humans by having smaller amounts of smooth muscle cells and elastic system fibers and larger amounts of collagen [27]. The smooth muscle of the rat's penis is located in the perisinusoidal region, while in man the smooth muscle is mixed with connective and elastic system fibers to form the trabeculae of the corpora cavernosa [28].

Although the rat penis presents these differences when compared to the penis of humans, several studies consider a suitable model to investigate the morphological changes resulting from pathological conditions [29, 30].

Vitamin D is an essential factor for a normal development; however, little is known regarding how vitamin D restriction during the perinatal and postnatal periods affects the growth of the offspring's penises. Therefore, the aim of this study was to evaluate the changes in morphology of the penis caused by restriction in vitamin D during the perinatal and postnatal periods in adult Wistar rats.

## 2. Material and Methods

The protocol was approved by the Ethics Committee for the Care and Use of Experimental Animals of the State University of Rio de Janeiro (Protocol CEUA/034/2014). The animals were placed in an environment with incandescent light, with no ultraviolet radiation, to prevent vitamin D synthesis in the skin.

Female Wistar rats, aged six weeks, were split into two groups: standard diet (SC, with vitamin D3, 0.25 g/kg, *n* = 8) and vitamin D restricted diet (VitD, without vitamin D3, 0.00 g/kg, *n* = 9). The diets were prepared in accordance with the nutritional recommendations for rodents by the American Institute of Nutrition (Table 1) [31].

The females had received the diets for six weeks before gestation, and, hence, vitamin D insufficiency or deficiency was achieved throughout gestation and lactation [32]. SC and VitD females were mated with males who received the SC diet. Females were fasting for 4 h for glucose assessment in the last gestational week [33]. An incision on the animal's tail was made, and its blood was obtained by milking. The measurement of serum glucose was obtained with a glucometer (Accu-Chek, Roche, São Paulo, SP, Brazil).

The diets were administered to the mothers until the end of the weaning. Body mass (BM) was evaluated weekly. Food and energy intake were recorded during pregnancy and lactation daily. Further, daily energy intake was estimated by multiplying the amount of feed intake in grams by the total energy of the diet in kilojoules.

The litter size at birth was randomly adjusted to six pups (three males and three females) per lactating mother to ensure adequate nutrition. The pups were divided into two groups: SC/SC (from SC mothers, fed SC diet from weaning (21 days) to 4 months of age, *n* = 8) and VitD/VitD (from VitD mothers, maintaining a weaning VitD diet to 4 months of age, *n* = 9).

The BM and nasoanal length were recorded weekly from birth to 4 months of age. Food and energy intake were monitored daily. The energy intake was calculated as previously described.

Systolic blood pressure (SBP) was measured weekly from the third to the fourth month of age, using plethysmography of the caudal artery (Insight, São Paulo, SP, Brazil).

At the end of the lactation period, euthanasia of mothers (*n* = 5 per group) was performed in a carbon dioxide gas chamber. Blood samples were collected from the right atrium for biochemical evaluations.

At birth, the pups (*n* = 5 per group) were sacrificed by decapitation and serum glucose was measured using a glucometer.

At 4 months of age, the offspring were fasted for 12 h, and they were placed in the carbon dioxide gas chamber. The blood samples were collected as previously described for biochemical analysis. A fasting blood glucose was measured with a glucometer (Accu-Chek, Roche, São Paulo, SP, Brazil); the penis was collected; and cross sections of the mid shaft were made to conduct histological procedures.

Following blood collection, plasma was separated by centrifugation (12.000 rpm for 15 min) at room temperature and stored at −80°C. The serum analyses of 25 hydroxyvitamin D3 (25 OHD3) and insulin were performed in duplicate by Enzyme-Linked Immunosorbent Assay (ELISA) (*n* = 8 animals per group). The 25 OHD3 serum level was evaluated with a CEA915Ge test kit (Cloud Clone Corp., Houston, Texas, USA). The serum insulin was analysed with a Rat/Mouse Insulin Kit (Rat/Mouse Insulin Kit, catalog, Millipore EZRMI13 K, St. Charles, Missouri, USA).

The samples of the mid shaft of the penis were fixed in 4% buffered formalin, and they were processed for being embedded in paraffin and 5 *μ* thick histological cuts were obtained. The cuts were stained with Weigert's resorcin-fuchsin technique with previous oxidation to evaluate the elastic system fibers. Masson's Trichrome was used to detect the connective tissue and sinusoidal spaces. The smooth muscle and cell proliferation were evidenced by immunohistochemical analysis using an anti-alpha smooth muscle actin and anti-proliferating cell nuclear antigen (PCNA) antibodies, respectively.

For immunohistochemistry stain, tissue sections were subjected to antigen retrieval with Tris-EDTA buffer, pH 9.0, overnight at 60°C. Endogenous peroxidase activity was blocked by incubating the slides with 3% H_2_O_2_ in methanol for 15 min followed by applying a protein block (10% goat serum at 60°C for 10 minutes). After draining this solution from the tissue section, the slides were incubated for 1 hour at 37°C with mouse monoclonal primary antibodies to alpha smooth muscle actin (18-0106, dilution 1 : 100, Invitrogen, Camarillo, USA) and PCNA (18-0110, dilution 1 : 100, Invitrogen, Camarillo, USA). Next, sections were treated with biotinylated secondary antibodies and the reaction was detected with the biotin-streptavidin-peroxidase complex (Kit Invitrogen, 859643, Frederick, USA) for 20 min and 3,3-diaminobenzidine tetrachloride (859643, Invitrogen, Frederick, USA) was used as the chromogen. After incubation, the sections were counterstained with Mayer's hematoxylin. Control tissue sections were obtained from the replacement of the primary antibody with 1% phosphate-buffered saline/bovine serum albumin. The stained tissues were observed with an Olympus BX51 optical microscope and photographed with an Olympus DP71 digital camera.

The cross sections of the penis were photographed with a Zeiss Axio Cam ERc5s camera in a Carl Zeiss optical microscope (Carl Zeiss, Gottingen, Germany). The following areas were measured: cross section of the penis (A), corpus cavernosum with tunica albuginea (CC with TA) and without tunica albuginea (CC without TA), tunica albuginea (TA), and corpus spongiosum (CS). The TA area was estimated by the difference between CC with TA and CC without TA. We evaluated 20 photomicrographs per animal and all quantifications were performed with a final magnification of ×18.9. These parameters were measured with the ImageJ software (Image Processing and Analysis in Java, NIH, Bethesda, Maryland, USA), with the freehand selection tool.

The percentages of connective tissue, smooth muscle, sinusoidal spaces, and elastic fibers were estimated using the point-counting method with a grid of 99 points superimposed on the magnified images using the grid tool of ImageJ software [34]. The cell counter tool of ImageJ software was used for counting separately each structure. The results were expressed as percentage. For each animal, 25 photomicrographs of the corpus cavernosum were obtained under ×400 magnification and 25 photomicrographs of the corpus spongiosum were captured at ×1000 magnification in different fields. To quantify the elastic fibers in corpus cavernosum were captured images at a final magnification of ×1000 and 25 fields per animal.

The immunostained nuclei with anti-PCNA antibody were quantified. The cell proliferation in the CC was quantified by the number of cells per mm^2^. The cell proliferation was measured at a final magnification of ×400 in 25 different fields per animal. The percentages and cell proliferation were estimated with the ImageJ software and the cell counter tool.

The digital images of the cuts were obtained using an Olympus DP70 camera (Tokyo, Japan) attached to an Olympus BX51 microscope (Tokyo, Japan).

Data were expressed as mean ± standard deviation. The differences between groups were evaluated by the unpaired Student's *t*-test. The level of significance was *p* < 0.05, and all analyses were conducted with the GraphPad Prism software, version 6.02, for Windows (GraphPad Software, San Diego, California, USA).

## 3. Results

No significant differences were observed in BM gain, energy intake, fasting glucose, and insulin in the mothers.

Serum levels of 25 OHD3 were significantly lower in VitD mothers (28.50 ± 2.20 ng/ml) compared with SC mothers (67.64 ± 12 ng/ml; *p* < 0.0001).

At the end of the experimental period, the BM were similar in animals fed with SC diet (414.20 ± 17.57 g) and VitD diet (432.60 ± 24.77 g). No difference with regard to food intake and energy was found between the offspring of both experimental groups. However, the animals in the VitD/VitD group showed a 15% increase in SBP at 4 months of age compared with the SC/SC group. An 87% increase in fasting blood glucose and 54% increase in serum insulin in relation to the SC/SC group were observed. In addition, the VitD/VitD group showed a reduction of 60% in the serum levels of 25 OHD3 compared with SC/SC. The data are presented in Table 2.

A 5% decrease of the cross-sectional area of the penis was found in the VitD/VitD compared with the animals in the SC/SC group. The CC areas with TA, and CC without TA were 7% and 6% lower in the VitD/VitD group compared with the SC/SC group, respectively. The TA area was 9% lower in the VitD/VitD group compared with the SC/SC group. Nevertheless, the area of the CS was 10% greater in the VitD/VitD group in relation to the SC/SC group (Table 2).

**Figure 1:**
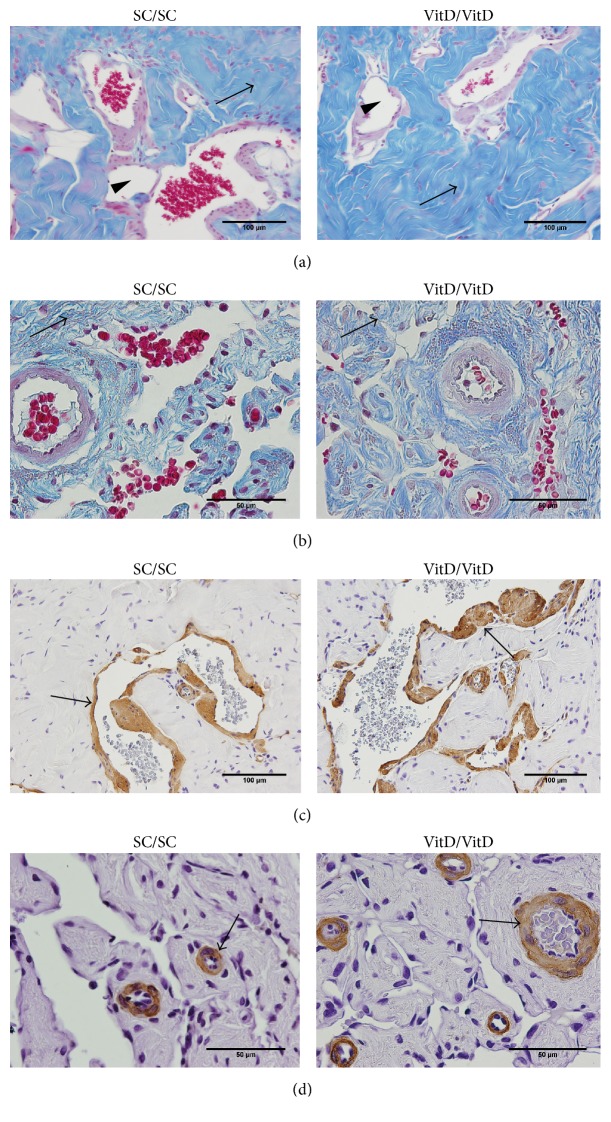
Photomicrographs of connective tissue, sinusoidal spaces, and smooth muscle in corpus cavernosum and corpus spongiosum of offspring penis at 4 months of age. (a) Corpus cavernosum; arrows indicate an increase of percentage of connective tissue and arrow head shows a reduction of sinusoidal spaces in VitD/VitD. Masson's trichrome, ×400. (b) Corpus spongiosum; arrows demonstrate an increase of percentage of connective tissue in VitD/VitD. Masson's trichrome, ×1000; arrows show an increased percentage of smooth muscle in corpus cavernosum (c) and in corpus spongiosum (d) in VitD/VitD. Immunostaining for anti-alpha smooth muscle actin, (c) ×400 and (d) ×1000, respectively.

The percentage of connective tissue increased 10% in the CC and 16% in the CS, in the VitD/VitD group compared with the SC/SC group (Figures 1(a) and 1(b), resp.).

**Figure 2:**
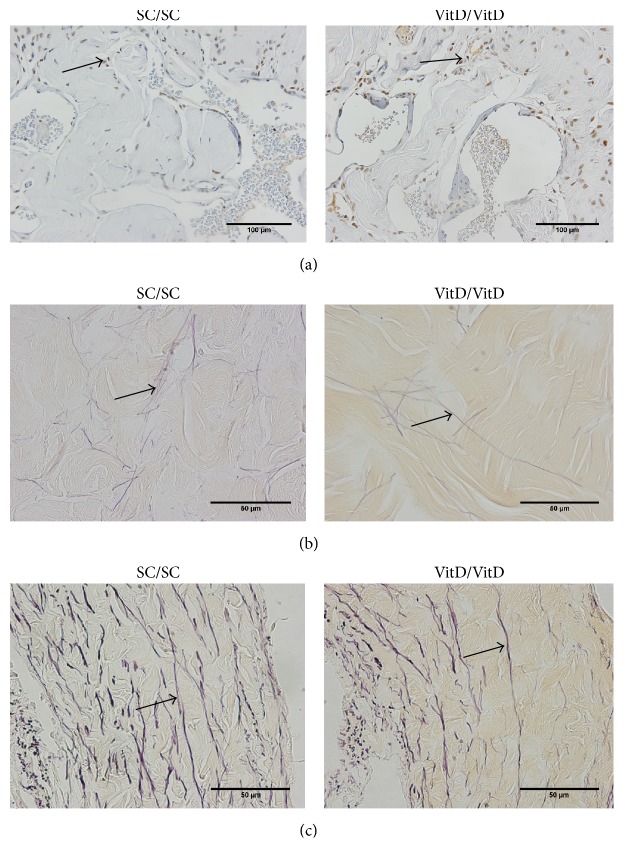
Photomicrographs of cell proliferation and elastic fibers in corpus cavernosum and corpus spongiosum of offspring penis at 4 months of age. (a) Corpus cavernosum; arrows show that the cell proliferation was greater in VitD/VitD. PCNA immunostaining, ×400. Arrows indicate a reduction of elastic fibers in (b) corpus cavernosum and (c) tunica albuginea of corpus spongiosum in VitD/VitD. Weigert's resorcin-fuchsin staining, ×1000.

The percentage of smooth muscle was 25% greater in the CC and 21% greater in the CS in the animals of the group VitD/VitD in relation to the SC/SC group (Figures 1(c) and 1(d), resp.). Nonetheless, the percentages of the sinusoidal spaces and elastic fibers in the CC of the animals in the VitD/VitD group were 21% and 15% lower, respectively, compared with the SC/SC group (Figures 1(a) and 2(b)).

The assessment of the TA of the CS revealed that the percentage of elastic fibers was 12% lower in the VitD/VitD group compared with the SC/SC group (Figure 2(c)). The data are presented in Table 2.

The cell proliferation was 17% greater in the CC of the animals in the VitD/VitD group in relation to the SC/SC group (Figure 2(a)).

## 4. Discussion

Vitamin D is stored in the liver and adipose tissue. In rodents, a six-week restriction leads to a significant reduction in their serum levels [35]. In this study, the VitD diet, consumed during six weeks before mating, during pregnancy, and during lactation, reduced the 25 OHD3 serum levels in the mothers. Consequently, their offspring were also affected, and they displayed a significant reduction of serum vitamin D levels. This was due to both the maternal diet and the diet received from weaning to 4 months of age.

The metabolic and histomorphologic alterations in the penis of adult offspring were consequences of vitamin D restriction in the perinatal and postnatal periods. This was due to the fact that no differences were observed in BM, glucose, insulin, or total energy intake between SC and VitD mothers. In addition, the BM, nasoanal length, and blood glucose of the offspring of both experimental groups were similar at birth.

Barassi et al. (2014) showed in a study that a significant proportion of ED patients have a vitamin D deficiency and low levels of vitamin D might increase the ED risk by promoting endothelial dysfunction [23]. Endothelial dysfunction plays an important role in pathogenesis of ED and vitamin D deficiency promotes endothelial dysfunctions [16]. The animals in the VitD/VitD group had fasting hyperglycemia, hyperinsulinemia, and elevated SBP. These metabolic changes are associated with endothelial vascular dysfunction, and they are risk factors for the onset of ED. This signals the importance of investigating the effects of vitamin D restriction on the morphology of the penis [36].

Vitamin D levels during pregnancy influence the fetal reserves. The offspring of mothers with hypovitaminosis D have deficiency of this vitamin during development [37]. Furthermore, testosterone levels decrease if there is vitamin D deficiency [38, 39]. Vitamin D metabolizing enzymes and receptors have been identified in the testes, indicating that vitamin D may play a role in regulating testosterone production [12]. The offspring that had low serum levels of vitamin D have, consequently, low serum levels of testosterone in adulthood.

Vitamin D restriction during the perinatal and postnatal periods resulted in a decrease in the A, CC with TA, CC without TA, and TA. A positive association was found between vitamin D levels and testosterone [40, 41].

Hofer and colleagues (2014) showed a possible action of vitamin D on the regulation of expression of steroidogenic genes and key enzymes to sex hormone biosynthesis [42]. Therefore, reduction of the A, CC with TA, CC without TA, and TA may be associated with vitamin D deficiency.

An increase in the amount of connective tissue in the CC and CS is related to fibrosis and ED [43]. The main event in the development of fibrosis is the increase in the expression of the transforming growth factor beta (TGF *β*) [43]. The animals of VitD/VitD group displayed an increase in SBP. Studies have shown that hypertension was associated with a decrease in elastic fibers and a thinning of the tunica albuginea in the penis of hypertensive animals which corroborates the results observed in the VitD/VitD group [44].

Since vit D reduces inflammatory and fibrogenic activity, vitamin D deficiency also led to an increase in fibrogenesis. This is related to an increase in TGF *β* expression, which is characterized by an increase in connective tissue in the CC and CS observed in the VitD/VitD group [45].

Fibroblasts are the most numerous cells in the CC of the rats, and the increase of the number of cells observed in the VitD/VitD is probably due to its proliferation [27]. Vitamin D is an antiproliferative hormone [7]. Restriction of this vitamin in the diet led to an increase in cell proliferation, which was observed in the VitD/VitD group. This corroborates an increase in the percentage of connective tissue in the CC and CS observed in animals of the VitD/VitD group.

Hyperglycemia has been observed in animals of the VitD/VitD group, and it induces glycosylation and degradation of elastin. This explains the decrease of elastic fibers in the VitD/VitD group [46, 47].

The decrease of elastic fibers in the CC and TA of the CS makes them less resistant to expansion during erection, reducing the pressure that causes ED [48]. Decrease in elastin in the penis of patients with severe ED has been documented in a previous study [49].

Vitamin D deficiency stimulates renin-angiotensin-aldosterone system, which may increase angiotensin II expression. This induces inflammatory response and vascular smooth muscle hypertrophy. In addition, vitamin D regulates the synthesis of endothelial nitric oxide synthase and nitric oxide (NO) [16]. Inadequate levels of vitamin D affect availability of NO. NO inhibits the growth of smooth muscle cells. This effect is mediated by the inhibition of proteins involved in regulation of the cell cycle [50]. These two factors explain the increase in area of the smooth muscles in the CC and CS found in the VitD/VitD group [16].

The growth of elements constituting the CC, such as the connective tissue and smooth muscle, may have resulted in a reduction of the area of the sinusoidal spaces in the VitD/VitD group. The changes in these elements lead to trabecular rigidity and alterations in the mechanical properties, which also result in ED [49].

## 5. Conclusion

Our study showed the influence of vit D as a remarkable micronutrient in the protection of penile cytoarchitecture. The results showed that vit D restriction in the perinatal and postnatal periods alters the penile morphology of adult offspring, indicating the importance of adequate serum levels of vit D during gestation, lactation, and postnatal life, to maintain the integrity of the penile morphology in the offspring.

## Figures and Tables

**Table 1:** Composition of the diets.

Nutrient (g/Kg)	SC AIN93G	VitD AIN93G	SC AIN93M	VitD AIN93M
Corn starch	397.48	397.48	465.69	465.69
Casein	200.00	200.00	140.00	140.00
Dextrinized starch	132.00	132.00	155.00	155.00
Sucrose	100.00	100.00	100.00	100.00
Soya bean oil	70.00	70.00	40.00	40.00
Fiber	50.00	50.00	50.00	50.00
L cystine	3.00	3.00	1.80	1.80
Choline	2.50	2.50	2.50	2.50
Antioxidant	0.014	0.014	0.008	0.008
Mineral mix	35.00	35.00	35.00	35.00
Calcium carbonate	357.00	357.00	357.00	357.00
Vitamin mix	10.00	10.00	10.00	10.00
Vitamin D3	0.25	0.00	0.25	0.00

All the nutrients corresponded to the recommendations of American Institute of Nutrition for rodents. SC, standard diet; Vit D, vitamin D restricted diet; AIN93G, diet for growth, pregnancy, and lactation; AIN93M, diet for adult maintenance.

**Table 2:** Parameters from offspring at 4 months of age.

Parameters	SC/SC	VitD/VitD	*p*
Food intake (g/animal/dia)	19.09 ± 3.98	18.91 ± 4.18	ns
Energy intake (kJ/animal/dia)	309.80 ± 62.46	306.30 ± 64.69	ns
Systolic blood pressure (mmHg)	164.00 ± 11.11	189.10 ± 8.08	<0.0001
Glucose (mmol/L)	6.04 ± 2.08	11.26 ± 4.15	0.0096
Insulin (*µ*UI/L)	21.50 ± 8.75	33.76 ± 3.52	0.0072
25 OHD3 (ng/ml)	73.71 ± 8.86	28.74 ± 0.20	<0.0001

Penile parameters	SC/SC	VitD/VitD	*p*

Morphometry			
A (mm^2^)	6.51 ± 1.35	6.15 ± 1.02	0.0083
CC with TA (mm^2^)	4.51 ± 0.95	4.19 ± 0.69	0.0007
CC without TA (mm^2^)	2.17 ± 0.54	2.05 ± 0.32	0.0133
TA (mm^2^)	2.34 ± 0.45	2.13 ± 0.45	0.0001
CE (mm^2^)	0.97 ± 0.12	1.02 ± 0.13	0.0015
Percentage in corpus cavernosum			
Connective tissue (%)	62.58 ± 13.81	68.54 ± 12.31	<0.0001
Smooth muscle (%)	8.55 ± 3.49	10.70 ± 3.70	<0.0001
Sinusoidal space (%)	22.24 ± 16.57	17.45 ± 8.33	0.0002
Elastic fibers (%)	5.09 ± 1.72	4.33 ± 1.93	0.0009
PCNA (cells/mm^2^)	4.63 × 10^−4^ ± 2.72 × 10^−4^	5.41 × 10^−4^ ± 2.58 × 10	0.0161
Percentage in corpus spongiosum			
Connective tissue (%)	59.04 ± 15.40	68.62 ± 14.79	<0.0001
Smooth muscle (%)	4.85 ± 2.61	5.87 ± 3.30	0.0074
Elastic fibers (%)	19.40 ± 9.42	19.73 ± 8.10	ns
Elastic fibers in tunica albuginea (%)	24.59 ± 6.79	21.00 ± 6.48	<0.0001

Data are presented as mean ± standard deviation and the differences were tested with an unpaired *t* test (*p* < 0.05). A, penile cross sectional area; CC with TA, corpus cavernosum area with tunica albuginea; CC without TA, corpus cavernosum area without tunica albuginea; TA, tunica albuginea; CE, corpus spongiosum; PCNA, proliferating cell nuclear antigen; ns, not significant.
